# Microbial Influence on the Mobility of +3 Actinides from a Salt-Based Nuclear Waste Repository

**DOI:** 10.3390/microorganisms11061370

**Published:** 2023-05-24

**Authors:** Julie Swanson, Adrianne Navarrette, Jandi Knox, Hannah Kim, Floyd Stanley

**Affiliations:** Los Alamos National Laboratory, Carlsbad, NM 88220, USA; anava@lanl.gov (A.N.); jknox@lanl.gov (J.K.); hannahyk@umich.edu (H.K.); floyd.stanley@srs.gov (F.S.)

**Keywords:** nuclear waste repository, bioassociation, neodymium, brine, salt, halophile

## Abstract

Biologically enhanced transport of radionuclides is one of several processes that can affect the performance of a nuclear waste repository. In this work, several microbial isolates from the Waste Isolation Pilot Plant (WIPP) were tested for their influence on the concentration of neodymium, as an analog for +3 actinides, in simple sodium chloride solutions and in anoxic WIPP brines. Batch sorption experiments were carried out over a period of 4–5 weeks. In many cases, the effect on neodymium in solution was immediate and extensive and assumed to be due to surface complexation. However, over time, the continued loss of Nd from the solution was more likely due to biologically induced precipitation and/or mineralization and possible entrapment in extracellular polymeric substances. The results showed no correlation between organism type and the extent of its influence on neodymium in solution. However, a correlation was observed between different test matrices (simple NaCl versus high-magnesium brine versus high-NaCl brine). Further experiments were conducted to test these matrix effects, and the results showed a significant effect of magnesium concentration on the ability of microorganisms to remove Nd from solution. Possible mechanisms include cation competition and the alteration of cell surface structures. This suggests that the aqueous chemistry of the WIPP environs could play a larger role in the final disposition of +3 actinides than the microbiology.

## 1. Introduction

Microorganisms can affect the mobility of actinides through various means, including, but not limited to, biosorption, biomineralization, and biologically induced precipitation. All of these processes are accounted for in the biocolloid enhancement parameters used to calculate the mobile actinide source term for the Waste Isolation Pilot Plant (WIPP) performance assessment. These parameters reflect the ability of microbiota to mobilize or immobilize radionuclides dissolved in the briny matrix of the WIPP repository.

The WIPP is the US repository for low- to intermediate-level, legacy nuclear waste. It is located in a subterranean, evaporite salt deposit—the Salado Formation. WIPP microbiology is unique in that it represents the intersection of hypersaline system ecology and repository ecology. As a result, microbial processes, such as biosorption and induced precipitation, that are of concern for repository performance may lead to different outcomes in salt, and the viability of organisms carrying out such processes might be constrained by such a high ionic strength environment.

Surface sorption is a metabolism-independent process in which radionuclides can bind to functional groups at a cell’s surface. In WIPP’s high ionic strength brines, the presence of competing cations (such as Na^+^, Mg^2+^, or Ca^2+^), at concentrations much higher than the radionuclides, can affect these surface interactions. Microorganisms can also promote ion supersaturation and subsequent precipitation, leading to further mineralization on or away from the cell’s surface. Again, brine ionic strength affects ion saturation, and brine constituents can compete with microbially generated ligands for mineral incorporation. Finally, these projected high ionic strengths (5–7 M) will dictate which, and how many, organisms can survive long-term in WIPP. This could limit the biomass available to sorb radionuclides.

Studies addressing the bioassociation of +3 actinides (or analogs) and lanthanides with halophilic microorganisms are limited but nevertheless point to both organism and matrix effects [1,2,3,4]. In one study, approximately 95% of the added americium (Am-EDTA) was removed by a pure halophilic bacterial culture isolated from WIPP environs, while a mixed culture of extreme halophiles (presumably archaea) removed only 28% [1]. The mechanism(s) of removal was not determined. In another study, increasing ionic strength led to increased Cm(III) and Eu(III) uptake by a halophilic bacterium, *Halomonas elongata* [2]. This was hypothesized to be due to an increase in cell surface hydrophilicity and changes in morphology, with a concomitant change in surface area at higher salt concentrations. Related experiments concluded that Cm(III) and Eu(III) sorption onto halophilic organisms (both bacteria and archaea) was lower than onto non-halophiles (bacteria), was less pH dependent, and occurred via exchange with Na^+^ ions at cell surface functional groups [3]. More recently, it was shown that exposure of *Halobacterium noricense*, an extremely halophilic archaeon, to Eu(III) resulted in both solid and aqueous Eu(III)-phosphate species, while Cm(III) interacted with surface carboxylate groups [4]. Finally, although not a halophile study, the effects of salt concentration were also observed in the interaction of the +3 analog, neodymium, with *Bacillus spores* [5]. In this work, salt-dependent release of the ligand dipicolinate led to Nd resolubilization rather than bioassociation.

Altogether, previous studies point toward the direct and indirect effects of high salt, or high ionic strength, on biological influence exerted by microorganisms. Direct effects include changes in radionuclide solubility and competition for surface sites from background electrolytes. Indirect effects are more related to the types of organisms that are present, especially the selection of halophilic organisms or cell surface characteristics.

This work was undertaken to look at possible trends in the bioassociation behavior of neodymium, as an analog for An(III), with several organisms isolated from the WIPP, and to determine the reasons for these trends, if observed. This information feeds directly into the WIPP performance assessment model as an input for biocolloid-induced transport of +3 actinides.

## 2. Materials and Methods

### 2.1. Solution Preparation

Several test solutions were prepared at NaCl concentrations matching the optimum for each organism (Supplementary Information, Table S1). These were adjusted to approximately pH 6 in order to ensure neodymium solubility, which varies with salt concentration [5]. Twice (2×) the target concentration of NdCl_3_·6H_2_O (Alfa Aesar, 99% purity) was added to each salt solution, and the pH was rechecked and readjusted, if necessary. All solutions were allowed to stand at least one week, in order to ensure no pH drift, and were then passed through a 0.2-micron filter to remove possible particulates.

WIPP brines were prepared anoxically at 90% of full strength (Table 1). Generic weep brine (GWB) is a high-magnesium brine that simulates interstitial fluids present at the WIPP. Energy Research and Development Administration Well 6 brine (ERDA-6; hereafter referred to as ERDA) is a high sodium chloride brine that simulates the composition of Castile Formation brine pockets that underlie the Salado. Brine pH was not adjusted, in order to simulate more realistic solution conditions. Neodymium was added to these brines from a concentrated stock (~2 mM) in 25% NaCl solution. The brines were allowed to stand for at least one week prior to filtration.

### 2.2. Organism Preparation

All test organisms were previously isolated from incubations of WIPP halite at various salt concentrations and had been putatively identified based on 16S rRNA gene sequencing [6,7]. With the exception of *Thalassobacillus* sp., each tested organism was grown to a stationary phase at its optimum NaCl concentration (Supplementary Information, Table S1). The cells were washed five times in NaCl solution matching the concentration of the growth media.

*Thalassobacillus* sp. was grown in broth at its optimum salt concentration and then flooded onto solid agar plates of matching composition. The plates were incubated for at least two weeks to ensure sporulation. Spores were harvested by scraping plates and purified by washing them five times with saline solution. Suspensions were checked by direct microscopy for the presence of vegetative cells and were not used if these were present. This spore purification method had been checked previously to ensure that no dipicolinic acid could be detected after the wash process.

### 2.3. Experimental Batch Setup

The final washed cell pellets were resuspended in their respective salt solutions to reach a target optical density corresponding to approximately 2 × 10^9^ cells/mL and then mixed 1:1 with the appropriate 2× Nd-NaCl solution. All organisms had been screened prior to the start of experiments to correlate optical density values and cell counts. The samples were then dispensed into triplicate 15 mL polypropylene centrifuge tubes and placed in a rotator. Negative controls were prepared without the microorganisms and at matching salt concentrations.

Experiments in WIPP brines were conducted in an oxygen-free, nitrogen-filled glove box. All batch tests were sampled over a period of at least one month. Experiments testing magnesium effects were carried out in a series of solutions with constant ionic strength (variable NaCl and variable MgCl_2_) and another series with variable ionic strength (constant NaCl and variable MgCl_2_; Supplementary Information, Table S2).

### 2.4. Sampling and Analysis

The samples were withdrawn from each tube periodically and measured for optical density using a GeneSys 50 spectrophotometer (ThermoFisher Scientific; Waltham, MA, USA). An aliquot of this was removed for direct cell counts using a Zeiss Axioscope 40 fluorescence microscope (ZEISS; Dublin, CA, USA) after staining with the LIVE/DEAD™ *Bac*Light™ nucleic acid stain (Invitrogen/Life Technologies; Carlsbad, CA, USA). The remainder of the sample was passed through an Amicon 100 kD centrifugal filter to remove biomass. A portion of the filtrate was diluted in 2% nitric acid containing an indium internal standard and analyzed for Nd concentration using an Agilent 7900 inductively coupled plasma mass spectrometer (Agilent Technologies; Santa Clara, CA, USA). Replicate filtrates were pooled for pH measurements.

The percentage of biologically influenced Nd was determined from the difference between the abiotic and biotic sample concentrations divided by the abiotic sample concentration. A higher percentage of influence indicates that more Nd was removed from the solution.

### 2.5. SEM-EDS Analysis

The samples were not fixed prior to examination by SEM in order to minimize the possible effects of the fixation process on precipitated solids. Even so, cells were visible for some organisms (*Nesterenkonia*, *Salinicoccus*, and *Thalassobacillus*). However, omitting fixation may have resulted in a negative effect on other organisms (haloarchaea and *Chromohalobacter*).

Approximately 50 µL of sedimented solids were aspirated from the bottom of the sample tubes and dispensed onto SEM sample stubs with carbon sticky tabs. The samples were allowed to air-dry and were then sputter-coated with gold (Ted Pella, Inc.; Redding, CA, USA). Visualization was carried out on a Phenom Pharos ProX/Nanoscience Instruments SEM (ThermoScientific; Waltham, MA, USA) at an acceleration voltage of 15 kV and equipped with energy dispersive X-ray spectroscopy (EDS) for elemental analysis.

## 3. Results

### 3.1. Tests in Simplified NaCl Solution

**Figure 1:**
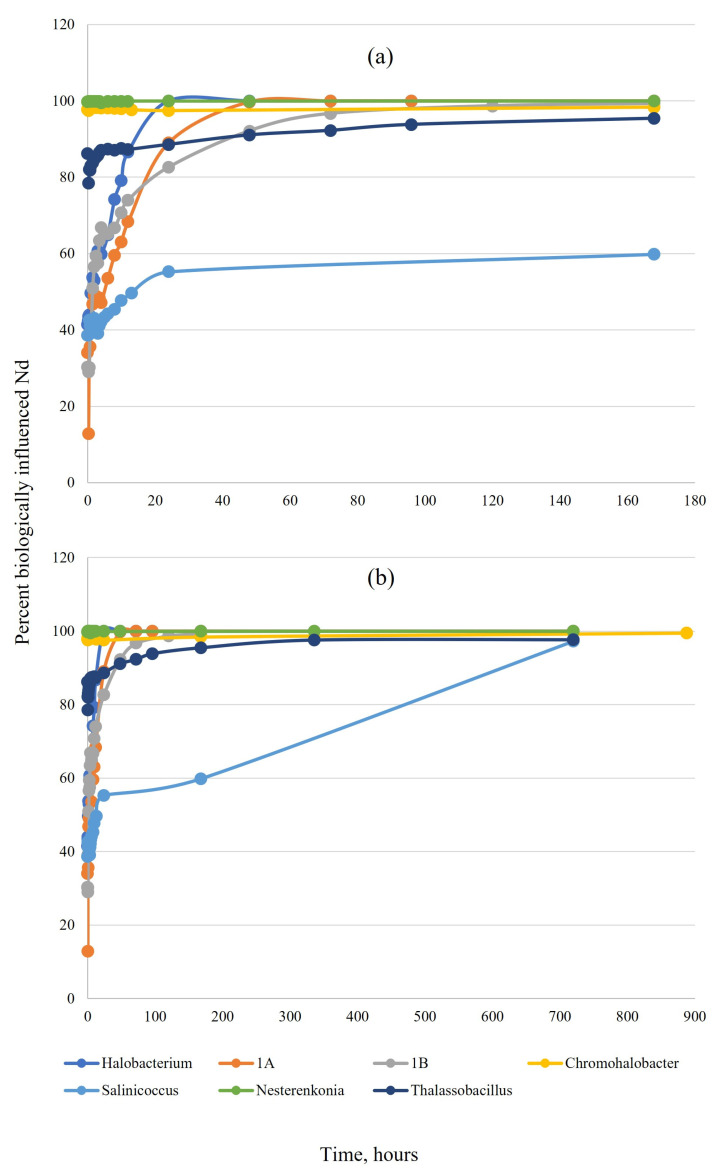
Comparison of the percentage of biologically influenced neodymium for all tested organisms in a simplified NaCl matrix over the course of one week (**a**) and one month (**b**). A higher percentage of influence indicates more Nd removed from the solution.

The initial amount of Nd lost from the solution varied with organism: *Nesterenkonia* sp. and *Chromohalobacter* sp. removed nearly 100% of Nd immediately upon exposure; *Thalassobacillus* spores removed 86%, and isolates 1A, 1B, *Saliniccocus*, and *Halobacterium* removed between 30–42% (Figure 1a). Over time, the percentage of biologically influenced Nd increased for all tested organisms and reached nearly 100% at one month, resulting in complete removal of Nd from the solution (Figure 1b).

#### 3.1.1. Effects of Biomass Concentration

**Figure 2:**
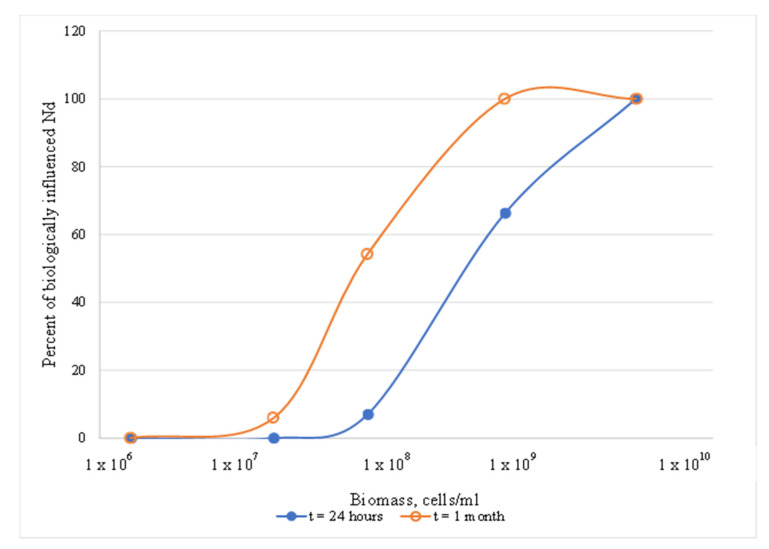
Effects of *Halobacterium* biomass concentration on Nd in NaCl solution.

Only *Halobacterium* was tested for biomass dependence. A significant increase in influence was observed at higher biomass concentrations in NaCl. This agrees with the literature and is logical, based on the increased number of available surface sites with increasing biomass. Over time, the percentage of influence increased, but only at biomass concentrations above 10^8^ cells/mL. In contrast, at lower (10^6^–10^7^ cells/mL) and higher (10^10^ cells/mL) cell densities, there was little to no change with time (Figure 2). Concentrations in actively growing cultures under ideal conditions can reach 5 × 10^9^ cells/mL, but ideal conditions are far from certain.

#### 3.1.2. Effects of NaCl Concentration

**Figure 3:**
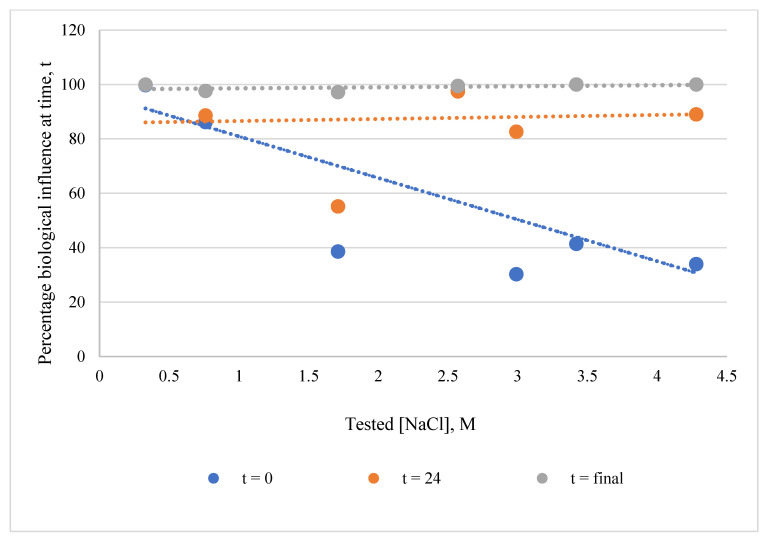
Correlation between the percentage of biological influence and matrix NaCl concentration at three time points. Each point within a series represents a different test organism.

Since each organism was tested at a different NaCl concentration to match its optimum, a comparison was made to see if NaCl concentration affected the organism’s influence. A weak negative correlation can be seen between the initial percentage of biological influence (regardless of organism) and the NaCl concentration in the simplified NaCl matrix. This suggests that immediate surface complexation could be subject to cation (Na^+^) competition (Figure 3). However, this correlation disappears completely over time, suggesting that in the long term, Nd disposition is controlled by a mechanism other than surface complexation.

### 3.2. pH Effects

The pH changed slightly in the abiotic NaCl samples (both decreasing and increasing), but an increase was observed in all the biotic NaCl samples (maximum 0.88 units). However, little change in pH was observed in the WIPP brines (Supplementary Information, Table S3).

### 3.3. Tests in WIPP Brines

**Figure 4:**
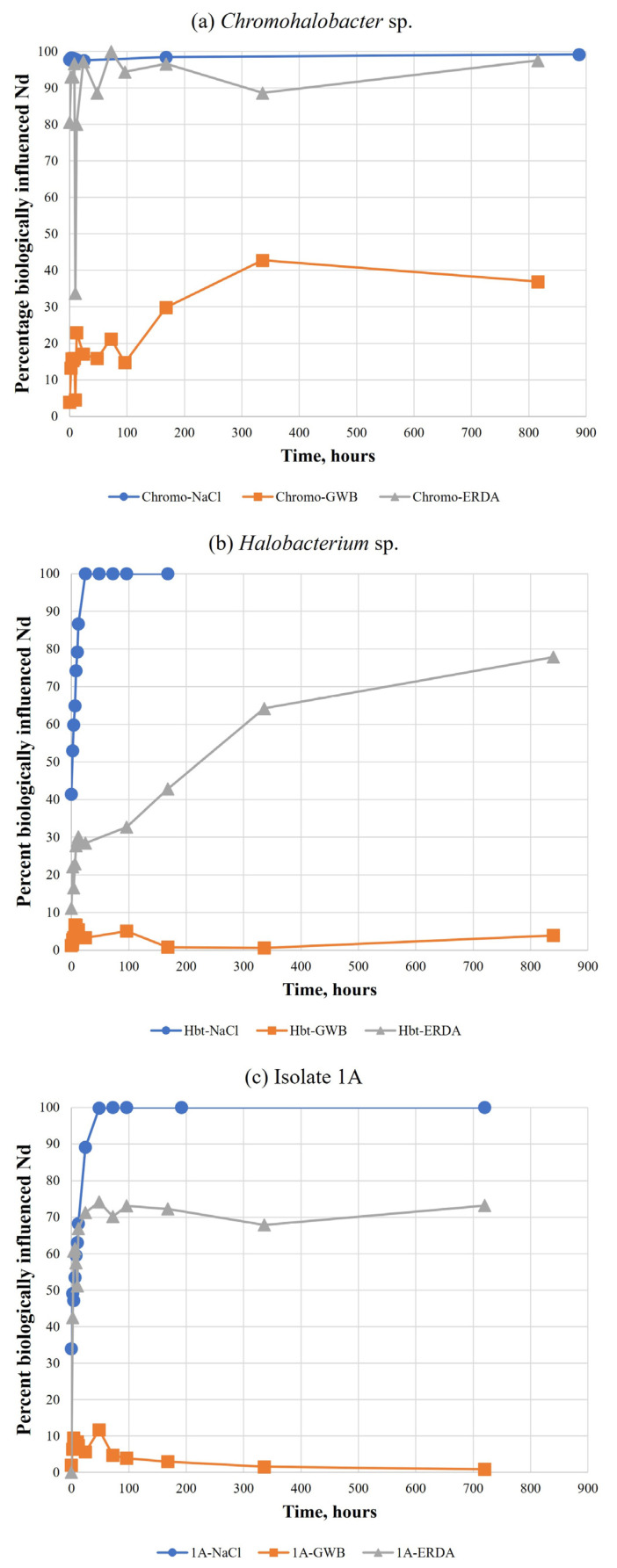
Comparison of the percentage of biologically influenced neodymium for three organisms tested in simple NaCl solution (blue circles), GWB (orange squares), and ERDA (gray triangles) over the course of approximately one month.

Three organisms—*Halobacterium* sp., isolate 1A, and *Chromohalobacter* sp.—were also tested in GWB and ERDA brines. In all three cases, the apparent association was much lower in GWB than in ERDA (Figure 4). In *Halobacterium* and isolate 1A investigations, influence in ERDA was less than simple NaCl (Figure 4b,c); for *Chromohalobacter*, NaCl and ERDA results were similar (Figure 4a).

### 3.4. Tests in MgCl_2_-NaCl Solutions

**Figure 5:**
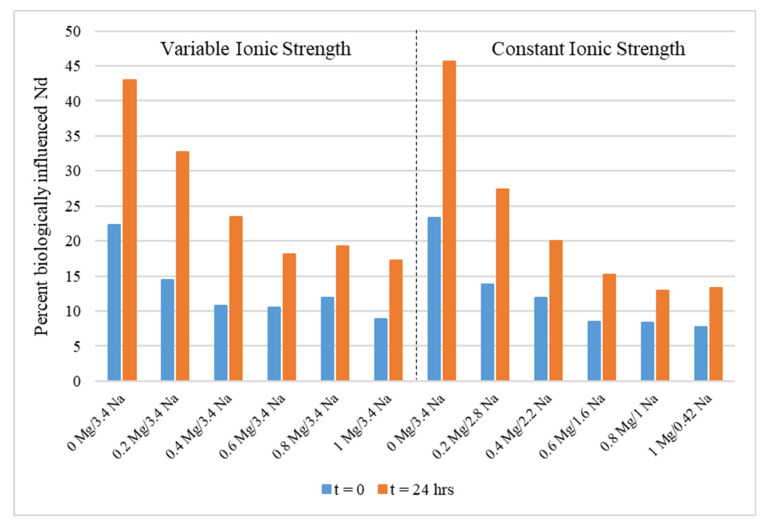
The effects of magnesium concentration on the percentage of biological influence on Nd in solution at variable and constant ionic strengths. Concentrations are given in M.

*Halobacterium* sp. was tested in a series of solutions with varying concentrations of NaCl and MgCl_2_. The results for these experiments clearly show decreasing biological influence as magnesium concentrations increase at early time points when surface complexation is likely to be the chief determinant of Nd loss from solution (Figure 5). The same trend was observed regardless of changes in sodium concentration and overall ionic strength.

### 3.5. SEM-EDS Analyses

#### 3.5.1. Precipitated Solids

**Figure 6:**
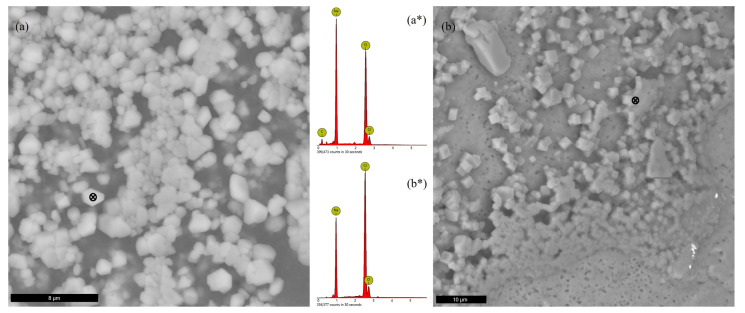
SEM images of precipitated solids from (**a**) NaCl solution (*Nesterenkonia* test solution) and (**b**) ERDA (*Halobacterium* test solution), along with associated EDS spectra (a*, b*) for marked spots.

**Figure 7:**
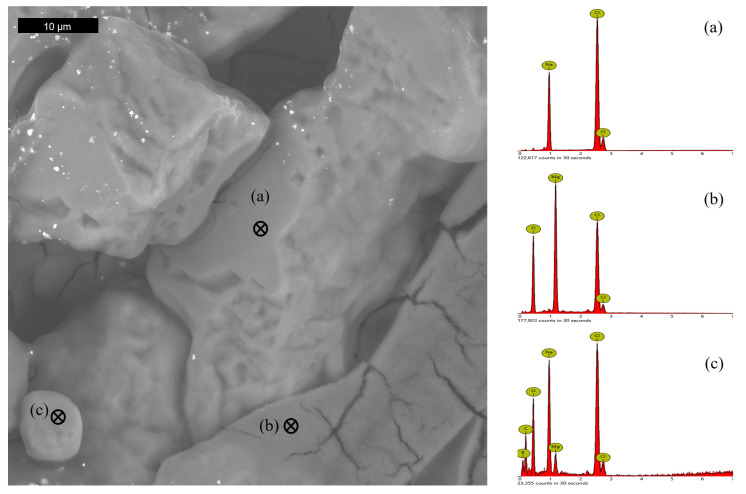
SEM image of precipitated solids from GWB (isolate 1A test) with associated EDS spectra for marked spots. Spot (**a**), NaCl; spot (**b**), magnesium-containing solid; spot (**c**), boron-containing solid.

Of the samples investigated at the end of the experiments, all revealed a significant amount of precipitated salts, regardless of the initial test matrix. Sodium chloride accounted for all the precipitates visible in that test matrix and for a majority in ERDA (Figure 6), while most of the solids precipitated from GWB contained magnesium (Figure 7). Neodymium was not detected by EDS in the examined images.

#### 3.5.2. Sedimented Biological Material

**Figure 8:**
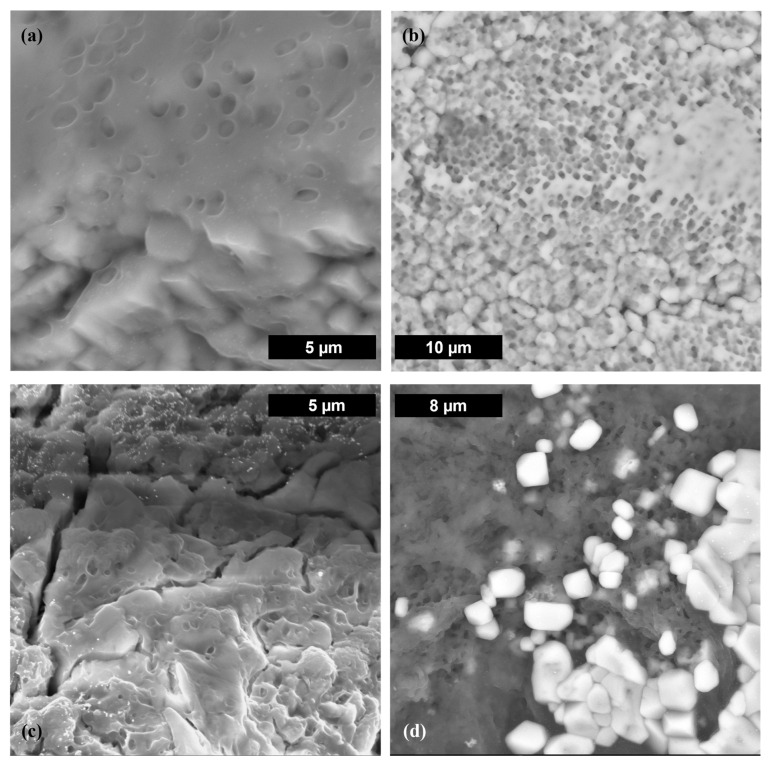
SEM images of exopolymeric structures (films and webs) formed by (**a**) isolate 1B, (**b**) *Salinicoccus*, (**c**) *Halobacterium*, and (**d**) *Chromohalobacter*, all in simple NaCl solutions.

*Nesterenkonia*, *Salinicoccus*, *Thalassobacillus,* and isolate 1A cells were present in large numbers, whereas no *Chromohalobacter*, *Halobacterium,* or isolate 1B cells were visible (all in NaCl test matrix). Omitting the fixation step is one possible reason for not seeing cells in those samples, but another reason could be cell loss during the actual experiments. This was verified, by microscopic cell counts, for *Thalassobacillus*, isolate 1B, and *Chromohalobacter* during the course of the experiments. In many samples (e.g., *Chromohalobacter*, isolate 1B, *Halobacterium*, *Salinicoccus*), amorphous organic material was observed in the form of a slimy matrix, opaque film, or weblike membrane. The material contained carbon, oxygen, nitrogen, sulfur, and phosphorus, suggesting extracellular polymeric substances (EPS) and possible biofilm formation (Figure 8).

## 4. Discussion

### 4.1. Comparison between Organisms

The six organisms used in this study are from two different domains of life (Bacteria and Archaea) and four different phyla (Euryarchaeota, Proteobacteria, Actinobacteria, and Firmicutes; Supplementary Material, Table S3). Three were Gram-negative, and three were Gram-positive, one of which was in spore form. Four are pigmented, but the pigments are not the same. All cells contain the same functional groups on their surfaces that can bind Nd, but in different densities and distributions.

The production of EPS and the presence of a glycoprotein surface layer (S-layer) were not determined experimentally here, but studies have found that two of these genera are known to produce EPS—*Salinicoccus* sp. and *Chromohalobacter* sp.—and three may possibly have S-layers—*Halobacterium* sp. and archaeal isolates 1A and 1B [8,9]. SEM-EDS images of four of the seven organisms (isolate 1B, *Halobacterium*, *Chromohalobacter*, and *Salinicoccus*) revealed exopolymeric materials. The decrease in *Chromohalobacter* cell numbers points toward cellular debris as a possible source of the organic material in these samples. *Salinicoccus* cells formed large clumps in all samples, possibly due to EPS. Not only would this result in a less available surface area, but it also caused sedimentation in the tubes. Given that Nd was not detected by EDS, it is not possible to know whether it was trapped within EPS or within a forming biofilm.

Nevertheless, these results highlight the variability between organisms at early time points (i.e., less than 24 h) but show that, over time, Nd loss from solution is not necessarily dependent upon organism type.

### 4.2. Comparison between Simple NaCl Solutions and WIPP Brines

The findings were similar for the three organisms that were tested in WIPP brines in addition to simplified NaCl solutions (*Halobacterium*, *Chromohalobacter*, and isolate 1A; Figure 4). Biological influence was highest in NaCl, followed by NaCl-based ERDA brine, while there was little to no influence of the organism on Nd concentration in GWB.

Because of the complex nature of the brine-microorganism suspensions, it is difficult to point to a single explanation for these findings. Indeed, there may be a combination of reasons or multi-modal interactions that account for our observations, including both chemical (speciation, cation competition) and biological (cell surface characteristics, mineralization) causes. Furthermore, these complex interactions can change with time, such that the initial cause of Nd loss from the solution may not be indicative of its final disposition.

#### 4.2.1. Neodymium Speciation

Experiments in simple NaCl solutions were conducted at ~pH 6 to minimize the effects of hydrolysis and carbonate. In those experiments, the majority of Nd (>95%) was present as Nd^3+^ and NdCl^2+^ with minor contributions from NdOH^2+^ (<4 %) and NdCl_2_^+^ (<1%) [5,10]. In experiments with WIPP brines, the pH was not adjusted. At these pH values (GWB: 7; ERDA: 8) and in the absence of carbonate, Nd is expected to exist as the hydrolysis species Nd(OH)_2_^+^ (>94%) and to a lesser extent NdOH^2+^ (2–5%) [5,11]. All these species are charged, suggesting that surface complexation is possible. In addition, borate species have been predicted to occur, and uncharacterized Nd-borate species have been detected in concentrated Mg solutions with added borate, although this species has low solubility [5,12].

Changes in pH can affect Nd speciation, so this parameter was tracked in all experiments. While changes were insignificant in the abiotic samples, the pH tended to increase in the biotic samples in the simple NaCl test solutions (Supplementary Information, Table S3). This resulted in an increase in the contribution of hydrolysis species from less than 5% to as much as 13% of the total Nd species (calculations in Geochemist’s Workbench; data not shown). However, in WIPP brines, the pH did not change relative to the abiotic controls, due to the buffering capacity of various brine constituents. Thus, speciation cannot explain the differences in apparent sorption between ERDA and GWB.

#### 4.2.2. Loss of S-Layers

The S-layer helps to maintain the cell structure in haloarchaea. Magnesium stabilizes S-layers, but changes in Mg^2+^ concentration or the presence of other chaotropic agents can lead to S-layer loss and subsequent changes in cell morphology [13]. Additionally, S-layer shedding or partial removal by vesicle formation is used by some organisms to remove toxic heavy metals [14,15,16,17,18].

The requirement for Mg^2+^ varies with the organism. For example, a *Haloarcula* species was found to change morphology at concentrations below 41 mM. The broth used to grow the organisms in this study contained 98 mM of Mg^2+^, whereas the brine formulations in these experiments contained 17 mM (ERDA) and 900 mM (GWB), suggesting that S-layer loss was possible in ERDA, due to low magnesium, but also possible in GWB, due to high magnesium.

Loss of the S-layer should have led to a decrease in surface binding sites, and a change in morphology to small coccoid cells would have resulted in less surface area overall. Ultimately, this should have resulted in less initial apparent sorption, regardless of brine. However, if the neodymium was bound in a shed and precipitated S-layer, this would appear as an increase in biological influence, since Nd would be lost from solution. Thus, it is possible that S-layer loss and precipitation can explain these observations, but only for organisms possessing this layer. This should be an area for further investigation.

#### 4.2.3. Magnesium Concentrations in Test Matrices

High concentrations of Mg can outcompete Nd due to the limited number of available binding sites at the cell surface. Investigations into the effects of magnesium on Nd-*Halobacterium* sp. interactions found that bioassociation decreases with increasing Mg (Figure 5). This trend appeared to be independent of ionic strength and sodium concentration above a threshold concentration of 0.6 M Mg, but was somewhat dependent on sodium content below this threshold.

The test GWB contained 0.9 M Mg, which suggests that cation competition was the reason for little to no observed bioassociation in those samples. ERDA contains less than 0.02 M Mg, which would explain why bioassociation occurred to a greater extent than in GWB, but to a lesser extent than in pure NaCl (Figure 4b,c). However, there was no difference between the association behavior in NaCl and ERDA samples with *Chromohalobacter* (Figure 4a). This could be due to an organism-specific difference, unrelated to cation competition. Cell numbers of *Chromohalobacter* dropped significantly over time in these experiments, especially in ERDA (from ~10^8^ to less than 10^5^ over ~5 weeks). Cell lysis could have left fragments and debris that were able to bind the neodymium, as has been shown with the cellular debris of other organisms and radionuclides or heavy metals [19]. In fact, SEM images of *Chromohalobacter* reveal the presence of such organic material (Figure 8d).

#### 4.2.4. Biologically Induced Formation of Nd-Containing Precipitates

Biologically induced mineral precipitation is a well-known phenomenon, and many reviews of the mechanisms of this process exist [20,21]. Microbial cell surfaces offer numerous potential nucleation sites, while microbial activity (even if minimal) can often alter solution characteristics, such as pH, localized ion concentrations, and organic releases that can promote mineralization [21]. In some cases, cell surface characteristics (e.g., EPS) enhance these conditions and can promote the initial nucleation step [22,23]. Furthermore, in high ionic strength matrices, it can be easier to reach the saturation index of specific ions. Biologically induced precipitation can result in the loss of Nd from solution but would have appeared as apparent sorption in these experiments.

Some haloarchaea have a high density of carboxyl sites at their surface that catalyze dolomite precipitation under ideal Mg:Ca ratios, even under non-growth conditions [24]. Members of the *Chromohalobacter* and *Nesterenkonia* genera can precipitate carbonate species, depending upon growth conditions [25,26], and some can incorporate strontium into the mineral phase, providing a possible radionuclide sink [27]. Resting cells of *Halobacterium noricense* DSM 15987^T^ sequestered europium and curium through the release of phosphate species, one of which was a solid-phase precipitate [4].

Some of the EDS analyses revealed the presence of phosphorus. Organisms can use phosphate complexation as a detoxification strategy to remove heavy metals. Complexation can be external, via release of phosphate into the surrounding environment or with phosphate functional groups on the cell surface, which can then lead to nucleation and mineralization of solids. In fact, both mechanisms were observed in experiments with *Halobacterium noricense* DSM 15987^T^, where both aqueous and solid Eu(III) phosphate species were detected [4]. Alternatively, phosphate sequestration can also be internal in the form of granules. Biologically precipitated minerals, such as hydroxyapatite, [Ca_5_(PO_4_)_3_OH], can incorporate +3 radionuclides into structures that are stable over time [24]. In many SEM images, precipitated solids were entrapped within the exopolymers (e.g., Figure 8a,b,d), suggesting that a solid Nd phase could likewise become immobilized.

In this study, SEM was not sensitive enough to detect Nd and would not have been able to differentiate between Nd bound to the cell surface or Nd in precipitated minerals at or near the cell’s surface. Additional analyses are planned to investigate this further.

### 4.3. Possible Internal Accumulation of Neodymium

Finally, it is possible that Nd was taken up into the cells. Internalization can result in either mobilization or immobilization, depending on the fate of the cells themselves. In these experiments, all cells settled out of suspension over time, forming a loose pellet at the bottom of the test tubes that was often associated with exopolymeric substances (WIPP performance assessment considers only those cells in suspension to be mobile).

## 5. Conclusions

These studies have shown that prokaryotic microorganisms can affect the dissolved concentration of the +3 analog, neodymium, in high ionic strength matrices. Simple surface complexation likely accounted for the initial removal of Nd from solution, but the long-term disposition of Nd is more complex. Several possible mechanisms exist for its biological removal: binding by the S-layer with subsequent shedding, precipitation, and possible mineralization; binding by cellular exopolymers with subsequent sedimentation, biofilm formation, and mineralization; internalization by cells with subsequent sedimentation. None of these possibilities could be definitively shown in this work, given the inability to detect Nd in the final samples. Future work will focus on additional analyses of solids (i.e., X-ray diffraction) and interactions on the cell surfaces.

Nevertheless, even if 100% of the added actinide is removed by cell surface complexation, the ultimate fate of the actinide depends on the fate of the cells themselves. Biosorption experiments rarely exceed 24 h, since their primary goal is to demonstrate the timely removal of metals for bioremediation strategies. For this reason, it is difficult to extrapolate the final disposition of a cell-bound radionuclide over an extended period of time.

That said, a repository’s performance is judged by its ability to contain waste radionuclides and prevent their release into the surrounding biosphere during its lifetime. The WIPP is mandated to account for a 10,000-year lifetime, but other repository concepts must account for much longer time frames—e.g., 10^5^ to 10^6^ years mandated for the Onkalo repository in Finland and proposed sites in Germany, respectively—depending upon the radioactivity of the emplaced waste.

Although these experiments are relatively short compared to a salt repository’s lifetime, they suggest that long-term biological influence on +3 actinides via association might be better predicted with knowledge of site matrix geochemistry rather than site microbiology. Repository-relevant magnesium concentrations appear to be inhibitory to bioassociation by simple surface complexation at initial timepoints, but may also result in significant changes to cell surface characteristics over the long term that lead to metal/actinide precipitation. This holds more true for GWB than ERDA-based brines, but it would be even more significant for the extremely high predicted Mg concentrations in the Asse II salt mine in Germany or in repository scenarios in which magnesium oxide is used as an engineered barrier. The idea that chemistry is a better predictor of +3 actinide (im)mobilization than microbiology is quite beneficial to performance assessments of salt-based repositories, as there is less uncertainty associated with the surrounding geochemistry than the microbiology. Investigations into mineral formation are ongoing.

## Figures and Tables

**Table 1:** WIPP brine compositions (90% of full strength).

Component	Concentration of Constituent, M	
	Generic Weep Brine (GWB)	ERDA-6 Brine
Na^+^	3.13	4.37
K^+^	4.14 × 10^−1^	8.73 × 10^−2^
Mg^2+^	9.01 × 10^−1^	1.71 × 10^−2^
Ca^2+^	1.22 × 10^−2^	1.08 × 10^−2^
Li^+^	3.89 × 10^−3^	NA
B_4_O_7_^2−^	3.49 × 10^−2^	1.42 × 10^−2^
Cl^−^	4.96	4.17
SO_4_^2−^	1.57 × 10^−1^	1.50 × 10^−1^
Br^−^	2.36 × 10^−2^	9.89 × 10^−3^

## Data Availability

The data presented in this study are either contained within the article and/or the supplementary material or available upon request from the Los Alamos National Laboratory—Carlsbad Operations Record Center through the corresponding author.

## Supplementary Materials

The following supporting information can be downloaded at: https://www.mdpi.com/article/10.3390/microorganisms11061370/s1, Table S1: Test organisms, growth media, and NaCl concentrations; Table S2: NaCl-MgCl_2_ test solutions for magnesium effects experiments; Table S3: pH measurements over time in biotic and abiotic samples; Table S4: Cell envelope characteristics possibly affecting biosorption.
