# Supplementary material for: Microbial Influence on the Mobility of +3 Actinides from a Salt-Based Nuclear Waste Repository
Source: Microorganisms. 2023 May 24;11(6):1370. doi: 10.3390/microorganisms11061370 (PMC10304748; doi:10.3390/microorganisms11061370)
Supplement: Supplementary file 1 [file microorganisms-11-01370-s001.zip › microorganisms-2398795-supplementary.pdf]

Supplementary Information

Table S1. Test organisms, growth media, and NaCl concentrations. All organisms were originally isolated from aerobic incubations of WIPP halite in GHB at the NaCl concentrations listed below.

Test Organism	Domain/Phylum	Growth Medium	[NaCl], M
Halobacterium (*noricense*)	Archaea/Euryarchaeota	GHB[1] + citrate	3.42
Archaeal isolate 1A	Archaea/Euryarchaeota	GHB	4.28
Archaeal isolate 1B	Archaea/Euryarchaeota	GHB	2.99
Chromohalobacter (*salexigens*)	Bacteria/γ-Proteobacteria	GHB	2.57
Salinicoccus (*roseus*)	Bacteria/Firmicutes	MB[2] + NaCl	1.71[3]
Nesterenkonia sp.	Bacteria/Actinobacteria	MB	0.33
Thalassobacillus (*cyri*) spores	Bacteria/Firmicutes	MB + NaCl	0.76[3]

[1] GHB = Generic Halophile Broth with variable NaCl concentrations [6]
[2] MB = Difco marine broth 2216 (Becton Dickinson)
[3] total of NaCl present in marine broth + NaCl added

Table S2. NaCl-$MgCl_2$ test solutions for magnesium effects experiments.

Solution	[$MgCl_2$], M	[NaCl], M	Ionic Strength, M
1V[1]	1	3.42	6.42
2V	0.8	3.42	5.82
3V	0.6	3.42	5.22
4V	0.4	3.42	4.62
5V	0.2	3.42	4.02
6V	0	3.42	3.42
1C[2]	1	0.42	3.42
2C	0.8	1.02	3.42
3C	0.6	1.62	3.42
4C	0.4	2.22	3.42
5C	0.2	2.82	3.42
6C	0	3.42	3.42

[1] V = variable ionic strength
[2] C = constant ionic strength

Table S3. pH measurements over time in biotic and abiotic samples (24 hours/1 week/~1 month).

Test Organism	In NaCl	
	Biotic	Abiotic
Halobacterium (*noricense*)	+0.65/+0.88/+0.77	+0.12/+0.04/-0.21
Archaeal isolate 1A	+0.32/+0.67/+0.76	+0.24/+0.44/+0.10
Archaeal isolate 1B	-0.08/+0.58/+0.40	-0.08/+0.10/-0.35
Chromohalobacter (*salexigens*)	+0.56/+0.65/+0.66	+0.02/-0.32/-0.96
Salinicoccus (*roseus*)	+0.26/+0.60/+0.36	-0.10/-0.28/-1.10
Nesterenkonia sp.	+0.44/+0.58/+0.82	-0.23/-0.20/-0.68
Thalassobacillus (*cyri*) spores	+0.44/+0.58/+0.82	-0.07/-0.41/-1.16
**Test Organism**	**In WIPP Brines**	
	**Biotic**	**Abiotic**
Halobacterium (*noricense*) in GWB	-0.17/-0.23/-0.18	-0.14/-0.34/-0.18
Halobacterium (*noricense*) in ERDA	-0.17/-0.25/-0.23	-0.14/-0.14/-0.21
Chromohalobacter (*salexigens*) in GWB	+0.25/+0.13/+0.17	+0.20/+0.13/+0.18
Chromohalobacter (*salexigens*) in ERDA	+0.16/+0.10/+0.12	+0.09/+0.11/+0.06
Archaeal isolate 1A in GWB	+0.12/-0.06/-0.01	+0.14/-0.04/+0.05
Archaeal isolate 1A in ERDA	+0.14/0.00/+0.05	+0.12/-0.01/-0.03

Table S4. Cell envelope characteristics possibly affecting biosorption.

Test Organism	Gram Stain Reaction	S-Layer[1]	EPS[2]	Pigment[3]
Halobacterium (*noricense*)	-	+	-	+
Archaeal isolate 1A	-	+	-	+
Archaeal isolate 1B	-	+	-	+
Chromohalobacter (*salexigens*)	-	-	+	-
Salinicoccus (*roseus*)	+	-	+	+
Nesterenkonia sp.	+	-	-	+
Thalassobacillus (*cyri*) spores	+	?	-	-

[1] surface layer; presence or absence is based on literature survey and not actual tests
[2] extracellular polymeric substances; presence or absence is based on literature and not actual tests
[3] pigments present are not necessarily the same type
